# Single-Gene Mutations in Hepatocellular Carcinoma: Applications and Challenges in Precision Medicine

**DOI:** 10.7150/ijms.117603

**Published:** 2025-07-10

**Authors:** Haiyang Yu, Xiangxiang Wu, Yiting Liu, Congling Xin, Yu Zhou, Xiaoyi Ding

**Affiliations:** 1 Department of Radiology, Ruijin Hospital, Shanghai Jiao Tong University School of Medicine, Shanghai 200025, China.; 2 Department of Radiology, Lianyungang TCM Hospital Affiliated to Nanjing University of Chinese Medicine, Lianyungang, 222004, China.; 3 Department of gynecology, Fudan University Shanghai Cancer Center Minhang District, Shanghai 200240, China.

**Keywords:** Hepatocellular carcinoma, HCC, Single-gene mutations, Precision medicine, Targeted therapy, Immunotherapy.

## Abstract

Hepatocellular carcinoma (HCC) is a genetically heterogeneous malignancy in which single-gene mutations serve as critical drivers of tumor initiation, progression, and therapeutic resistance. Advances in high-throughput genomics and liquid biopsy technologies have highlighted the clinical utility of mutations in genes such as TP53, CTNNB1, and TERT as diagnostic, prognostic, and predictive biomarkers. These mutations disrupt key oncogenic pathways, modulate the tumor immune microenvironment, and contribute to intratumoral heterogeneity, complicating disease management. Mutation-guided precision medicine, including telomerase inhibitors, Wnt/β-catenin pathway modulators, and immune checkpoint blockade, offers promising avenues for individualized treatment in HCC. However, challenges persist in translating these findings into clinical practice due to mutation complexity, resistance mechanisms, and limitations in biomarker standardization. Emerging strategies such as multi-omics integration, artificial intelligence, and gene editing technologies hold potential to overcome these barriers and facilitate the development of personalized therapeutic regimens. This review summarizes the molecular mechanisms, clinical applications, and translational challenges of single-gene mutations in HCC, with the aim of guiding future research and precision oncology.

## Introduction

Hepatocellular carcinoma (HCC) is the most common primary malignancy of the liver and has been witnessing a steadily increasing incidence worldwide. Major etiological factors include chronic viral hepatitis (particularly hepatitis B and C), liver cirrhosis, and metabolic syndrome 1. In recent years, advances in molecular biology and genomics have elucidated the critical roles that single-gene mutations play in the initiation and progression of HCC. These mutations not only alter key biological properties of tumor cells but also represent potential therapeutic targets, offering new avenues for precision medicine 2,3.

Among the genetic alterations associated with HCC, mutations in TP53 are the most frequently observed and are closely linked to tumor initiation, progression, and prognosis 4. TP53 mutations lead to dysregulation of the cell cycle and may impair antitumor immune responses within the tumor microenvironment, thereby influencing patient outcomes 5. Other key mutations, such as those in CTNNB1 and AXIN1, contribute to hepatocarcinogenesis by modulating key signaling pathways 6. The advent of single-cell sequencing technologies has enabled high-resolution analysis of the genomic, transcriptomic, and epigenomic landscapes of HCC. This approach facilitates the identification of intratumoral heterogeneity and rare cellular subpopulations, offering insights into their roles in disease progression and revealing novel therapeutic targets 2,7,8.

In the context of precision medicine, single-gene mutation profiling provides promising biomarkers for early diagnosis and prognostic assessment of HCC, thereby laying the foundation for individualized treatment strategies. For instance, specific mutations may predict patient response to immune checkpoint inhibitors and guide clinical decision-making 9,10. Additionally, liquid biopsy technologies enable the detection of circulating tumor DNA (ctDNA) mutations, allowing for real-time monitoring of tumor dynamics and the optimization of personalized therapeutic regimens 11,12.

In summary, research on single-gene mutations in HCC has significantly enhanced our understanding of its pathogenesis and propelled the development of precision oncology. With continued advancements in genomic profiling and personalized therapeutic approaches, there is great potential to improve long-term outcomes and quality of life for patients with HCC.

## Pathogenic Mechanisms of Single-Gene Mutations in HCC

Single-gene mutations serve as critical pathogenic drivers in the initiation and progression of HCC. These mutations not only play a direct role in tumorigenesis but also contribute to disease progression by modulating key signaling pathways and exacerbating tumor heterogeneity 4-6. This section will provide a focused discussion on three major aspects: (i) the most common types of driver gene mutations in HCC, (ii) the mechanisms by which these mutations lead to aberrant activation of signaling pathways, and (iii) the potential impact of single-gene mutations on intratumoral heterogeneity. These insights aim to deepen our understanding of the molecular pathogenesis of HCC and support the development of targeted precision therapies.

### Common Driver Gene Mutations in HCC

Driver gene mutations involved in the initiation and progression of HCC can be broadly categorized into three groups: tumor suppressor gene mutations, oncogene mutations, and mutations in epigenetic regulatory genes. Among tumor suppressors, TP53 is the most frequently mutated gene in HCC. The p53 protein encoded by TP53 plays a pivotal role in cell cycle regulation and apoptosis. Approximately 30% of HCC patients harbor TP53 mutations, which impair the cellular response to DNA damage and thereby promote tumorigenesis 4,13. Mutations in other tumor suppressor genes such as AXIN1 and RB1 are also closely associated with HCC. AXIN1 mutations activate the Wnt/β-catenin signaling pathway, enhancing cellular proliferation, while RB1 regulates the G1/S cell cycle checkpoint. Loss of RB1 function disrupts cell cycle control and contributes to malignant transformation 14-16.

In terms of oncogenes, mutations in CTNNB1 are frequently observed in HCC and similarly lead to aberrant activation of the Wnt/β-catenin pathway, driving tumor development. Additionally, mutations in the TERT promoter region activate telomerase, extending cellular lifespan and facilitating malignant transformation of hepatocytes 5,14,17,18.

Mutations in epigenetic regulatory genes also play significant roles in HCC pathogenesis. Alterations in ARID1A and ARID2 disrupt chromatin remodeling, affecting gene expression and cellular proliferation. Mutations in MLL3 and KMT2D (also known as MLL2) lead to aberrant histone methylation patterns, contributing to epigenetic dysregulation and tumorigenesis 9,18,19.

### Aberrant Activation of Key Signaling Pathways

The pathogenesis of HCC is strongly associated with the dysregulation of several key signaling pathways. Among these, the Wnt/β-catenin signaling pathway is one of the most frequently altered. Its activation is often driven by mutations in CTNNB1, resulting in enhanced cell proliferation and tumor formation 14. Another critical pathway is the p53/MDM2 axis; overexpression of MDM2 promotes the degradation of p53, thereby inhibiting its tumor-suppressive function and facilitating carcinogenesis 4.

In addition, aberrant activation of the Mitogen-Activated Protein Kinase (MAPK) and PI3K/Protein kinase B (AKT)/mTOR pathways is closely linked to HCC progression. These pathways promote cell growth, survival, and proliferation, contributing significantly to the malignant behavior of HCC cells 5,20.

### Impact of Single-Gene Mutations on HCC Heterogeneity

Single-gene mutations not only contribute to the initiation of HCC but also play a crucial role in shaping tumor heterogeneity. Spatial and temporal heterogeneity of genetic mutations results in distinct biological behaviors among different tumor cell subpopulations, which may underlie therapeutic resistance and disease recurrence 2,22. Studies have shown that HCCs harboring different mutation profiles exhibit marked differences in clinicopathological characteristics. For instance, HCCs with CTNNB1 mutations are often associated with higher tumor grades and poorer prognoses 5,23. A deeper understanding of how single-gene mutations influence tumor heterogeneity is essential for the development of effective, personalized treatment strategies.

## Applications of Single-Gene Mutations in Precision Medicine for HCC

**Figure 1:**
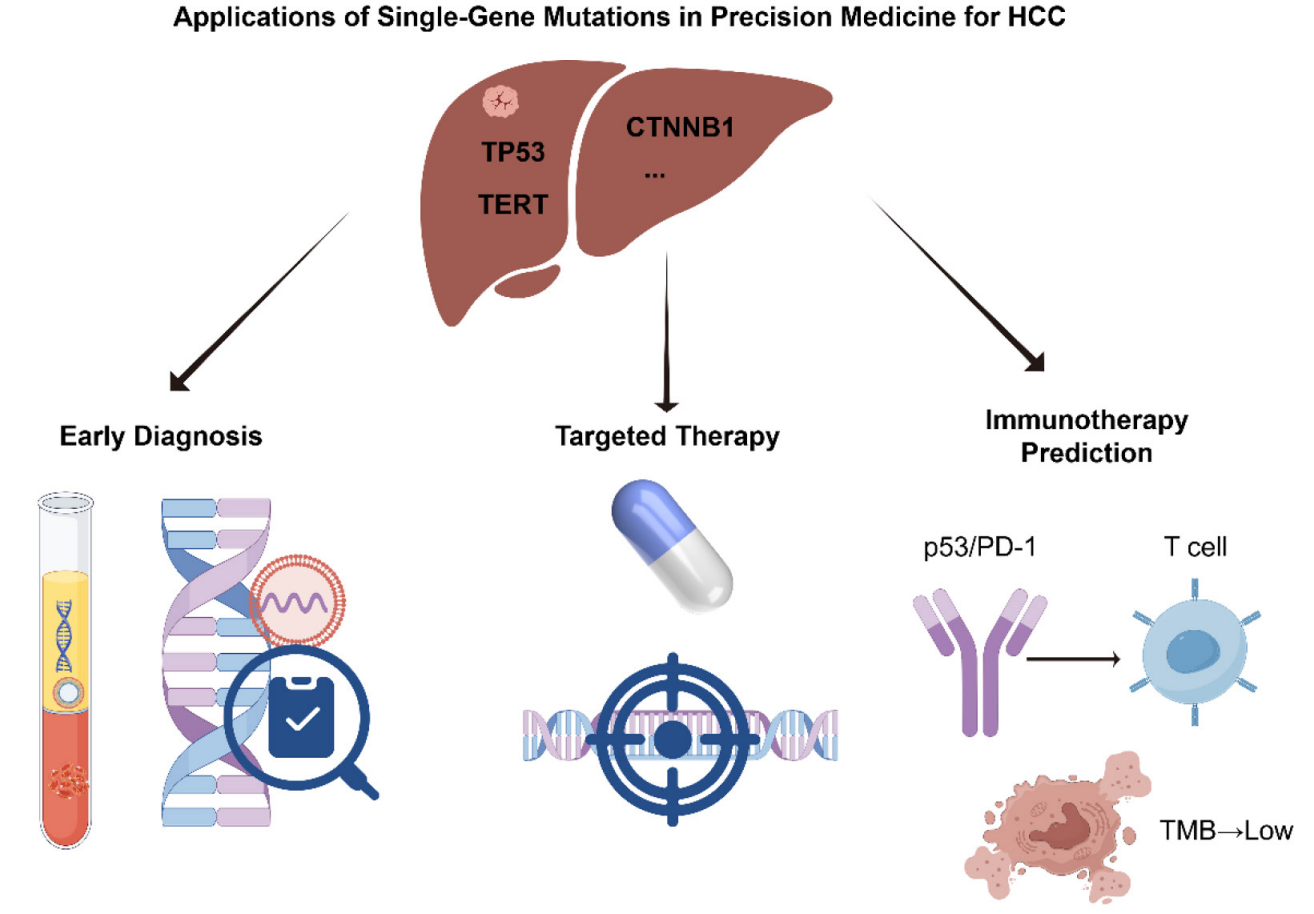
Applications of Single-Gene Mutations in Precision Medicine for HCC (by Figdraw).

With the growing understanding of the molecular mechanisms underlying HCC, the clinical value of single-gene mutations in precision oncology has become increasingly evident 24,25. Specific genetic alterations not only play critical roles in HCC initiation and progression but also serve as potential biomarkers for early diagnosis and provide theoretical and practical guidance for targeted and immunotherapeutic strategies 26,27. In recent years, significant advances have been made in mutation-informed liquid biopsy technologies, the development of targeted therapies, and the prediction of immunotherapy responses, offering more precise and individualized treatment options for HCC patients 28,29. This section provides a comprehensive overview of the multifaceted applications of single-gene mutations in HCC precision medicine, with a focus on early diagnosis, targeted therapy, and immunotherapy (Figure 1).

### Application of Single-Gene Mutations in Early Diagnosis of HCC

Early diagnosis of HCC is crucial for improving therapeutic outcomes and patient prognosis. However, due to the asymptomatic nature of early-stage disease, traditional imaging and serological markers often lack sufficient sensitivity and specificity. As a result, molecular diagnostic approaches based on single-gene mutations have emerged as promising tools for early HCC detection 30. In particular, mutations in driver genes such as TERT and CTNNB1 have become focal points of recent research. Concurrently, advances in liquid biopsy technologies have greatly enhanced the feasibility and accuracy of early HCC detection 31.

Studies have shown that TERT promoter mutations occur at a high frequency in HCC and are closely associated with tumorigenesis, making them valuable molecular markers for early diagnosis and prognostic assessment 32. Mutations in CTNNB1 are often linked to aberrant activation of the Wnt/β-catenin signaling pathway and are useful for identifying molecular subtypes and tracking disease progression in HCC 33. The detection of these key mutations in blood samples enables the identification of high-risk individuals prior to the onset of clinical symptoms, providing a foundation for early intervention and precision disease management 34.

The development of liquid biopsy has introduced new opportunities for the non-invasive diagnosis of HCC. Circulating tumor DNA (ctDNA) reflects the mutational landscape of tumor tissues and has been shown to reliably detect various HCC-related mutations, including those in TERT and CTNNB1 35,36. In addition, extracellular vesicles (EVs) containing microRNAs (miRNAs) have emerged as promising biomarkers. For instance, miR-122 levels are significantly reduced in the EVs of HCC patients, and restoration of its expression has been shown to suppress tumor progression, highlighting its potential value in early HCC detection 37,38. Liquid biopsy technologies offer the advantages of being minimally invasive and clinically accessible, providing an effective means of capturing tumor-specific genetic information. These approaches hold great promise for the early detection and individualized treatment of HCC.

### Targeted Therapy Guided by Single-Gene Mutations

As research into the molecular mechanisms of HCC has progressed, the identification of specific driver gene mutations has opened new avenues for personalized therapy. Therapeutic strategies targeting these mutations aim to improve treatment efficacy and enhance patient outcomes.

#### TERT Promoter Mutations and Telomerase Inhibitors

Mutations in the promoter region of the TERT (telomerase reverse transcriptase) gene are among the most common genetic alterations in HCC. These mutations lead to increased telomerase activity, enabling unlimited proliferation of tumor cells 39. Consequently, the development of telomerase inhibitors has become a key area of research. Preclinical studies have demonstrated that these inhibitors can effectively suppress HCC cell proliferation and delay tumor progression in animal models 40,41. However, such agents remain in the preclinical stage, and their efficacy and safety in humans require validation through large-scale clinical trials.

#### CTNNB1 Mutations and Wnt/β-Catenin Pathway Inhibitors

The CTNNB1 gene encodes β-catenin, a central component of the Wnt signaling pathway. Mutations in CTNNB1 result in aberrant activation of Wnt/β-catenin signaling, thereby driving HCC development and progression 14. Inhibitors targeting this pathway—such as small molecules and monoclonal antibodies—have shown tumor-suppressive potential in preclinical studies. However, since the Wnt pathway also plays essential roles in normal physiological processes, off-target effects and toxicity have limited their clinical application. Several of these inhibitors are currently undergoing early-phase clinical trials to evaluate their safety and therapeutic potential 42,43.

#### FGFR4 Mutations and FGFR Inhibitors

Fibroblast growth factor receptor 4 (FGFR4) is frequently overexpressed or mutated in HCC, contributing to increased tumor invasiveness and metastatic potential 44. FGFR inhibitors, particularly tyrosine kinase inhibitors (TKIs), have been evaluated in both preclinical models and clinical trials. For HCC patients with aberrant FGFR4 signaling, these inhibitors exhibit promising antitumor activity and may improve clinical outcomes 44-46.

#### Other Potential Targeted Therapies

In addition to the above mutations, other molecular targets are actively being investigated for their therapeutic potential in HCC. For instance, vascular endothelial growth factor (VEGF) and its receptors play critical roles in angiogenesis. Anti-VEGF agents such as sorafenib and lenvatinib have already been approved for first-line treatment of advanced HCC 47.

### Genetic Mutation Biomarkers in Immunotherapy

With the expanding use of immune checkpoint inhibitors (ICIs) in HCC treatment, identifying biomarkers that predict immunotherapeutic efficacy has become a key focus in precision immuno-oncology. Recent studies have highlighted the role of specific gene mutations in modulating the tumor immune microenvironment and influencing response to ICIs. Among these, mutations in TP53, CTNNB1, and tumor mutation burden (TMB) are the most representative and well-studied 48-50.

TP53 is one of the most frequently mutated genes in HCC. The protein it encodes, p53, plays a critical role in maintaining genomic stability and regulating apoptosis 48,51. Loss-of-function TP53 mutations not only drive tumorigenesis but also contribute to immune evasion by upregulating PD-L1 expression and impairing antitumor immune responses. These mutations are associated with decreased T cell activity and poor response to immunotherapy, suggesting that TP53 status may serve as a potential biomarker for predicting ICI sensitivity 48,51.

Mutations in CTNNB1, on the other hand, impact the immune microenvironment primarily through activation of the Wnt/β-catenin signaling pathway 14. Mutant CTNNB1 leads to a significant reduction in T cell infiltration, resulting in a so-called “cold tumor” phenotype that is inherently resistant to ICIs. HCC patients harboring CTNNB1 mutations typically exhibit poor responses to immunotherapy, further underscoring the negative predictive value of this alteration 52-54.

Additionally, tumor mutation burden (TMB) has emerged as a pan-cancer biomarker for assessing immunotherapy efficacy and is increasingly being investigated in the context of HCC 55,56. Although the overall TMB in HCC is relatively low compared to other cancer types, subsets of patients with high TMB tend to respond better to PD-1/PD-L1 blockade and have more favorable prognoses 55,57. Thus, TMB may also hold promise as a predictive biomarker for immunotherapy in HCC.

Despite these promising developments, translating mutation-informed insights into clinical practice remains fraught with challenges.

## Challenges of Single-Gene Mutations in Precision Medicine

Despite significant advancements in the molecular characterization of HCC and the development of precision medicine strategies, translating these findings into clinical practice remains challenging. On one hand, the genetic landscape of HCC is highly complex, involving not only cooperative interactions among multiple genes but also numerous rare single-gene mutations, which limit the broad applicability of targeted therapies 3,4,58. On the other hand, clinical implementation of precision medicine is hindered by factors such as drug resistance, inter-individual genetic heterogeneity, and the limited stability of biomarkers 3,48,59,60. Furthermore, although emerging technologies such as liquid biopsy show considerable promise, their diagnostic performance and clinical validation require further refinement. While targeted and immunotherapeutic interventions have shown favorable outcomes in selected clinical cohorts, their real-world efficacy and consistency with mutational profiles remain to be fully elucidated. This section discusses key challenges currently facing HCC research, focusing on the complexity of genetic mutations, barriers to clinical translation, and the application of molecular biomarkers.

### Complexity of Single-Gene Mutations

HCC is a genetically heterogeneous malignancy characterized by intricate networks of gene-gene interactions and mutation combinations. Common driver mutations, such as those in TP53 and CTNNB1, often co-occur and may synergistically affect cellular proliferation, apoptosis, and metabolic pathways, thereby accelerating tumor progression 4,61. These combinatorial mutations can also influence therapeutic efficacy; for instance, certain mutations may confer resistance to targeted therapies, complicating treatment planning and response prediction 3.

In addition, a substantial number of rare genetic mutations have been identified in HCC, the functional characterization of which remains a significant challenge. Due to their low prevalence and limited clinical data, it is often difficult to obtain sufficient cases for large-scale functional validation 10. Although such rare mutations may play critical roles in specific subsets of patients, their biological relevance and clinical significance require further investigation and verification.

### Challenges in the Clinical Translation of Single-Gene Mutations in Precision Medicine

The clinical application of precision medicine in HCC faces several translational hurdles. One of the most critical limitations is the development of resistance to targeted therapies. Although initial responses to treatment are often favorable, tumor cells frequently acquire resistance through mechanisms such as secondary mutations, signaling pathway reprogramming, or alterations in the tumor microenvironment, ultimately leading to therapeutic failure 62. In addition, pronounced inter-population heterogeneity in mutation profiles significantly limits the universal applicability of precision treatment strategies 63,64. Genetic mutations can vary in type and frequency depending on factors such as ethnicity, geographic origin, and environmental exposure, all of which influence drug response and prognosis 63,64. These issues underscore the need for deeper investigation into resistance mechanisms and the establishment of population-specific mutation databases to enable truly individualized therapeutic approaches.

### Reliability and Standardization of Single-Gene Mutations as Biomarkers

As a non-invasive approach, liquid biopsy has shown promise in the early detection and dynamic monitoring of HCC. However, it still faces technical limitations in sensitivity and specificity, particularly in early-stage tumors where false-negative rates remain high, compromising diagnostic accuracy 65,66. Moreover, most existing studies are based on single-center cohorts with small sample sizes, lacking large-scale, multi-center clinical validation. This hampers the broad adoption and clinical integration of liquid biopsy in routine practice 65-68. Therefore, improving the analytical performance of liquid biopsy platforms and establishing standardized clinical workflows through multi-center trials are crucial next steps to enhance the reliability and translational impact of mutation-based biomarkers in HCC.

## Future Directions in Single-Gene Mutation Research

**Figure 2:**
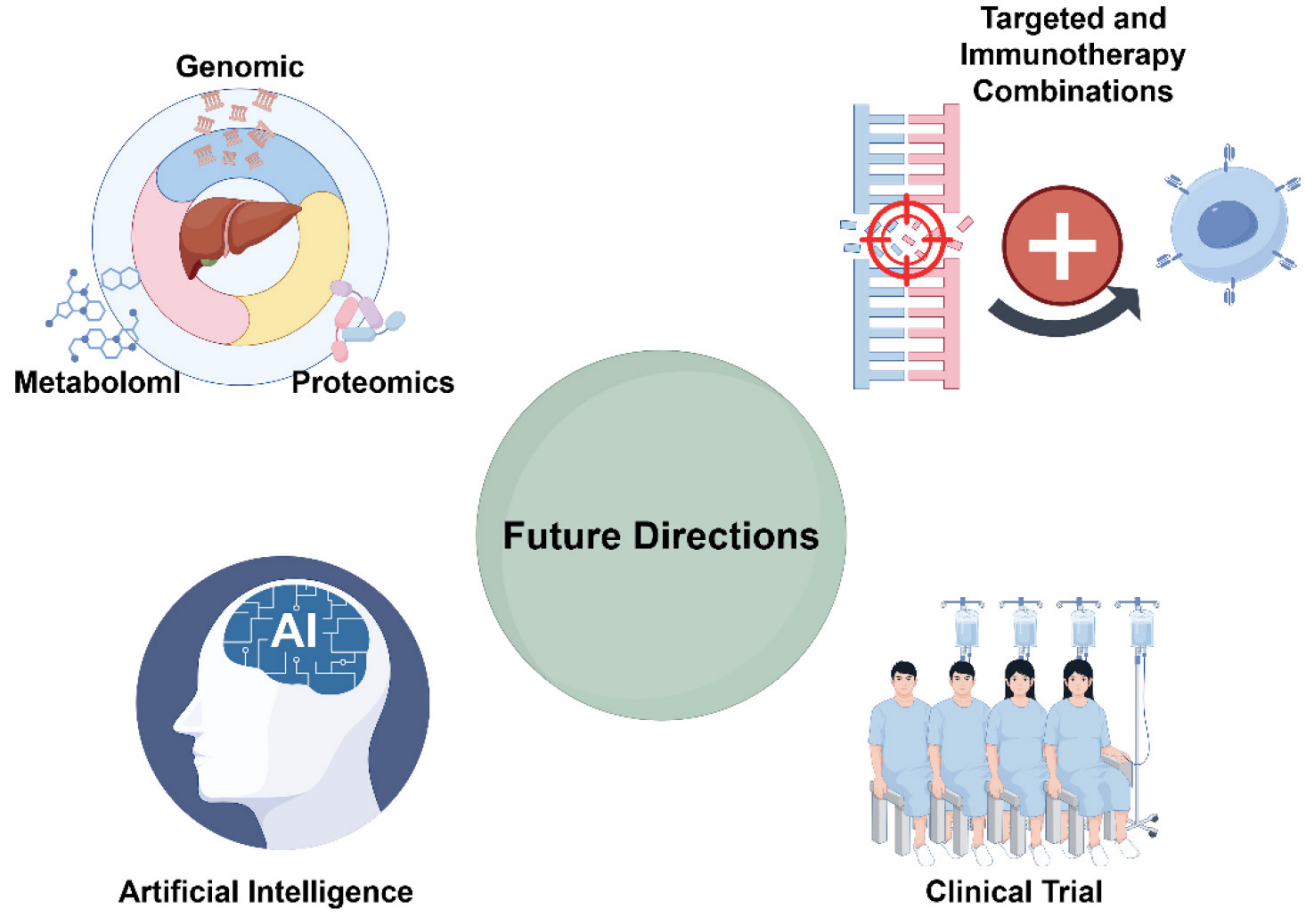
Future Directions in Single-Gene Mutation Research (by Figdraw).

As understanding of the molecular mechanisms underlying HCC continues to deepen, future research must not only adopt more sophisticated technologies and multidimensional data integration but also focus on bridging therapeutic strategies with clinical translation 69,70. This section explores the latest advances and prospective directions in HCC research—from multi-omics integration and AI-assisted precision medicine to novel therapeutic strategies and clinical trial design—providing a theoretical foundation and strategic insights for advancing personalized medical practice (Figure 2).

### Integrated Multi-Omics Analysis

Integrated multi-omics analysis is becoming increasingly vital in HCC research. By systematically combining genomic, transcriptomic, proteomic, and metabolomic data, researchers can comprehensively elucidate the molecular mechanisms, tumor heterogeneity, and progression dynamics of HCC. This approach facilitates the identification of key driver genes, signaling pathways, and potential therapeutic targets, enabling the construction of regulatory networks centered on single-gene mutations. Such networks help uncover core oncogenic drivers and regulatory axes that may guide therapeutic interventions 71-73.

The rapid development of single-cell omics technologies has made it possible to perform high-resolution genomic, transcriptomic, and epigenetic analyses of HCC tissues at the single-cell level 74,75. This has greatly advanced our understanding of intratumoral heterogeneity, dynamic changes in the immune microenvironment, and the roles of rare cellular subpopulations. These insights provide a robust theoretical basis for precision subtyping and individualized treatment strategies.

### Artificial Intelligence

With the widespread application of artificial intelligence (AI) and big data analytics in biomedical research, HCC studies are entering a new era of innovation. AI technologies—particularly deep learning and graph neural networks—are capable of efficiently processing large-scale omics datasets, clinical variables, and imaging data to uncover latent biomarkers and predictive models 76. In recent years, AI has been increasingly employed in critical aspects of HCC research, including drug response prediction, immune response profiling, and prognostic modeling. For instance, by training models on multi-omics datasets correlated with treatment outcomes, AI can identify patient subgroups that are more likely to benefit from immune checkpoint inhibitors or targeted therapies, thereby enhancing the precision of therapeutic decision-making 77. Additionally, AI can support intelligent patient stratification and adaptive design in clinical trials, improving their efficiency and scientific rigor 78. Looking ahead, the deep integration of multi-omics data with AI-driven algorithms is expected to yield high-throughput, high-precision, and interpretable disease models for HCC, substantially accelerating the translation of basic research into clinical applications and advancing the implementation of precision medicine in HCC management.

### Emerging Strategies in Targeted and Immunotherapy Combinations

Innovative combinations of targeted therapy and immunotherapy are becoming a central focus in HCC treatment research. The Clustered Regularly Interspaced Short Palindromic Repeats (CRISPR) / Cas9 gene-editing system, with its high efficiency and specificity, offers a novel avenue for molecular therapy in HCC 79. This technology allows for precise targeting and modification of oncogenes that are critically involved in HCC progression, thereby effectively suppressing tumor cell proliferation, invasion, and metastasis 79. Recent studies have shown that CRISPR not only serves as a powerful tool for gene function validation and elucidation of oncogenic mechanisms but also holds promise in combination with immunotherapies—such as immune checkpoint inhibitors—to modulate the tumor immune microenvironment and enhance the antitumor efficacy of immune cells 79,80.

Moreover, patient responses to immune checkpoint blockade in HCC are highly heterogeneous, with a subset of individuals exhibiting immune tolerance or evasion 81. Prior studies suggest that mutations in key driver genes such as TP53 and CTNNB1 can influence tumor immune phenotypes and therapeutic sensitivity 4. Therefore, stratifying patients based on their mutational profiles and designing personalized immunotherapeutic regimens may improve clinical outcomes and reduce immune-related adverse events. The integration of gene-editing technologies with immune checkpoint inhibitors not only expands the therapeutic arsenal against HCC but also provides a robust theoretical and technical foundation for precision and individualized interventions.

### Clinical Trial Design and Implementation

In designing clinical trials for HCC, accounting for patient heterogeneity and tumor complexity is essential 81. Flexible trial frameworks, such as adaptive trial designs, allow for real-time modifications to treatment strategies based on interim results, thereby enhancing trial efficiency and relevance 82. Moreover, integrating multi-omics data and AI-driven predictive modeling at the design phase can optimize patient selection and tailor treatment regimens accordingly 83. Ensuring patient engagement and adherence during trial implementation is also critical for success. Researchers must design protocols that are patient-friendly and minimize barriers to participation, thus improving enrollment and compliance 84. Collectively, these strategies can significantly advance clinical research in HCC and broaden access to effective, personalized treatment options.

## Conclusion

Research on single-gene mutations in HCC plays a pivotal role in elucidating disease mechanisms, identifying biomarkers, and advancing personalized therapeutic strategies. The pathogenic effects of different mutations vary across populations, shaped by the interplay between genetic background and environmental factors. Integrated multi-omics analysis offers a powerful approach to dissect the complex biology of HCC and to uncover novel therapeutic targets. Future efforts should focus on bridging basic scientific discoveries with clinical application, thereby accelerating the implementation of precision medicine in the diagnosis and treatment of HCC.

To achieve this goal, greater emphasis should be placed on multidisciplinary collaboration, innovative clinical trial designs, and international data sharing frameworks. These strategies will be essential for overcoming translational barriers and ensuring the real-world applicability of mutation-informed precision oncology in HCC.
